# Supplementary material for: District level correlates of COVID-19 pandemic in India during March-October 2020
Source: PLoS One. 2021 Sep 30;16(9):e0257533. doi: 10.1371/journal.pone.0257533 (PMC8483309; doi:10.1371/journal.pone.0257533)
Supplement: S1 Fig — Source: Author’s calculations. (DOCX) [file pone.0257533.s001.docx]

**S1 Figure. Trend of bi-weekly total confirmed cases in India, 14 March –November 5, 2020**

Source: Author’s calculations
