# Supplementary material for: District level correlates of COVID-19 pandemic in India during March-October 2020
Source: PLoS One. 2021 Sep 30;16(9):e0257533. doi: 10.1371/journal.pone.0257533 (PMC8483309; doi:10.1371/journal.pone.0257533)
Supplement: S1 Table — Source: Author’s Computation. (DOCX) [file pone.0257533.s002.docx]

**S1 Table Average number of new cases, recovered, infected, deceased cases bi-weekly (14 days) in India (March 14, 2020 – November 5, 2020)**

| Biweekly/Average | New Cases | Infected | Recovered | Deceased |
| --- | --- | --- | --- | --- |
| 14-27 March | 63 | 63 | 5 | 1 |
| 28 March - 10 April | 479 | 536 | 51 | 16 |
| 11-24 April | 1204 | 1616 | 336 | 38 |
| 25 April - 8 May | 2517 | 3347 | 885 | 86 |
| 9-22 May | 4648 | 6194 | 2425 | 124 |
| 23 May - 5 June | 7959 | 10058 | 4385 | 209 |
| 6-19 June | 11396 | 14761 | 7213 | 452 |
| 20 June - 3 July | 18147 | 21878 | 12865 | 407 |
| 4-17 July | 27898 | 32773 | 18554 | 544 |
| 18-31 July | 46900 | 55700 | 31533 | 734 |
| 1-14 Aug | 59155 | 73788 | 50858 | 898 |
| 15-28 Aug | 66820 | 74218 | 59999 | 970 |
| 29 Aug -11 Sept | 85440 | 91290 | 69565 | 1056 |
| 12 Sept- 25 Sept | 88888 | 103706 | 87491 | 1136 |
| 26sept-9Oct | 76805 | 77066 | 81393 | 1003 |
| 10-23 Oct | 59755 | 54165 | 73409 | 753 |
| 24 Oct- 5 Nov | 42663 | 28256 | 53647 | 502 |

Source: Author’s Computation.
