# Supplementary material for: District level correlates of COVID-19 pandemic in India during March-October 2020
Source: PLoS One. 2021 Sep 30;16(9):e0257533. doi: 10.1371/journal.pone.0257533 (PMC8483309; doi:10.1371/journal.pone.0257533)
Supplement: S2 Table — Source: Author’s Computation. Note: Computation of Infected Cases Ii = (Ci-Ri-Di). (DOCX) [file pone.0257533.s003.docx]

**S2 Table State-wise COVID-19 cases in India (14^th^ March - 31^st^ October, 2020)**

| States | Confirmed Cases | Infected Cases | Recovered Cases | Deceased Cases | Population Projected 2020 | IR per one hundred thousand Population |
| --- | --- | --- | --- | --- | --- | --- |
| INDIA | 8182676 | 571096 | 7489426 | 122154 | 1332808756 | 42.85 |
| Andaman & Nicobar Island | 4328 | 178 | 4091 | 59 | 397000 | 44.84 |
| Andhra Pradesh | 823348 | 24575 | 792083 | 6690 | 52221000 | 47.06 |
| Arunachal Pradesh | 14852 | 1856 | 12959 | 37 | 1504000 | 123.40 |
| Assam | 206249 | 9264 | 196051 | 934 | 34293000 | 27.01 |
| Bihar | 216764 | 7863 | 207811 | 1090 | 119520000 | 6.58 |
| Chandigarh | 14418 | 641 | 13551 | 226 | 1179000 | 54.37 |
| Chhattisgarh | 187270 | 22090 | 163079 | 2101 | 28724000 | 76.90 |
| Dadar & Nagar Haveli | 3246 | 80 | 3164 | 2 | 586956 | 13.63 |
| Daman & Diu | 0 | 0 | 0 | 0 | 586956 | 0.00 |
| Delhi | 386706 | 32719 | 347476 | 6511 | 19814000 | 165.13 |
| Goa | 43626 | 2344 | 40678 | 604 | 1540000 | 152.21 |
| Gujarat | 172943 | 13005 | 156220 | 3718 | 67936000 | 19.14 |
| Harayan | 167210 | 12192 | 153229 | 1789 | 28672000 | 42.52 |
| Himachal Pradesh | 22059 | 2908 | 18839 | 312 | 7300000 | 39.84 |
| Jammu & Kashmir | 94785 | 6419 | 86888 | 1478 | 13203000 | 48.62 |
| Jharkhand | 101736 | 5277 | 95575 | 884 | 37403000 | 14.11 |
| Karnataka | 823412 | 55028 | 757209 | 11175 | 65798000 | 83.63 |
| Kerala | 433106 | 91302 | 340319 | 1485 | 35125000 | 259.93 |
| Ladhakh | 6270 | 656 | 5539 | 75 | 293000 | 223.89 |
| Lakshadweep | 0 | 0 | 0 | 0 | 64473 | 0.00 |
| Madhya Pradesh | 171359 | 8928 | 159479 | 2952 | 82232000 | 10.86 |
| Maharashtra | 1678406 | 124143 | 1510353 | 43910 | 122153000 | 101.63 |
| Manipur | 18502 | 3472 | 14862 | 168 | 2855794 | 121.58 |
| Meghalaya | 9404 | 1110 | 8206 | 88 | 3224000 | 34.43 |
| Mizoram | 2722 | 430 | 2291 | 1 | 1192000 | 36.07 |
| Nagaland | 9010 | 1602 | 7375 | 33 | 2150000 | 74.51 |
| Odisha | 290116 | 12994 | 275749 | 1373 | 43671000 | 29.75 |
| Puducherry | 35015 | 3699 | 30724 | 592 | 1504000 | 245.94 |
| Punjab | 133658 | 4257 | 125198 | 4203 | 29859000 | 14.26 |
| Rajasthan | 196993 | 15102 | 179984 | 1907 | 77264000 | 19.55 |
| Sikkim | 3953 | 349 | 3536 | 68 | 610577 | 57.16 |
| Tamilnadu | 724522 | 22164 | 691236 | 11122 | 75695000 | 29.28 |
| Telangana | 238122 | 17899 | 218887 | 1336 | 37220000 | 48.09 |
| Tripura | 30714 | 1516 | 28855 | 343 | 3992000 | 37.98 |
| Uttar Pradesh | 481863 | 23768 | 451070 | 7025 | 224979000 | 10.56 |
| Uttarakhand | 62328 | 4382 | 56923 | 1023 | 11141000 | 39.33 |
| West Bengal | 373664 | 36886 | 329937 | 6841 | 96906000 | 38.06 |

Source: Author’s Computation. Note: Computation of Infected Cases I_i_= (C_i_-R_i_-D_i_)
