# Supplementary material for: District level correlates of COVID-19 pandemic in India during March-October 2020
Source: PLoS One. 2021 Sep 30;16(9):e0257533. doi: 10.1371/journal.pone.0257533 (PMC8483309; doi:10.1371/journal.pone.0257533)
Supplement: S3 Table — Source: Infection Ratio (IR) from provided data from COVID- 19 Dashboard of India. Independent variables computed from Census of India (2011) and fourth round of National Family Health Survey (IIPS;2017); **mortality estimates (reference period five year of the preceding. (DOCX) [file pone.0257533.s004.docx]

**S3 Table Brief description of each indicator used in this analysis**

| Indicator | Description of variables |
| --- | --- |
| Infection Ratio per lakh population | Number of confirmed cases of COVID -19 in the ith district based on per lakh population |
| Demographic variables | |
|  | Age |
| Total population 60+ | Percent of 60+population |
| Working Population | Percent of population 15-59 population |
|  | Migration |
| Marginal Worker | Percent of marginal worker |
|  | Density |
| Population density | Area per 1000 population |
| Socio-economic variables | |
|  | Education |
| Literacy Rate | Percent of Literate population |
|  | Caste |
| SC population | Percent of Schedule caste population |
| ST population | Percent of Schedule tribe population |
|  | Religion |
| Hindu population | Percent of Hindu population |
|  | Place of Residence |
| Urban Area | Percent of the population residing in urban area. |
| Average room size | Average person sleeping in the room |
|  | Household Amenities |
| Soap availability | Percent of household with soap availability |
| Water availability | Percent of households with water availability within premises |
| Toilet facility | Percent of the household having toilet facility within premises |
| Woman morbidity status | |
| Diabetes (Glucose>140mg) | Percent of women having glucose level >140mg |
| Woman morbidity (self-reported) | |
| Cancer Disease cancer among 18+women | Percent of women who 18+ self-reported cancer |
| Tobacco consumption among 18+woman | Percent of women aged 18+ consumption of tobacco |
| Health status variables | |
| Testing ratio | Ratio of testing population per lakh |
| Under-5 mortality rate | Probability of death under age five per 1000 live births |
| Institutional births | Percent of women having institution delivery |
| Full immunization | Percent of children received full immunization |
| Anemia among women | Percent of women aged 18+ who reported anemia |
| Stunting among children | Percent of children who have stunting |
| Wasting among children | Percent of children who have wasting |

Source: Infection Ratio (IR) from provided data from COVID- 19 Dashboard of India. Independent variables computed from Census of India (2011) and fourth round of National Family Health Survey (IIPS;2017); **mortality estimates (reference period five year of the preceding
