# Supplementary material for: District level correlates of COVID-19 pandemic in India during March-October 2020
Source: PLoS One. 2021 Sep 30;16(9):e0257533. doi: 10.1371/journal.pone.0257533 (PMC8483309; doi:10.1371/journal.pone.0257533)
Supplement: Attachment — Submitted filename: Review_PLOSONE_34338.docx [file pone.0257533.s005.docx]

The study on “District level correlates of COVID-19 pandemic in India” about exploring the hot and cold spots in terms of districts and finding the socio-economic, demographic and health-related determinants of COVID-19 disease in India. The study uses publicly available data from COVID19 India API. The study uses descriptive statistics of COVID-19 disease and linear regression model using indicators from NFHS-4 and Census of India to establish the findings. The outcomes of the study are based on three major findings from: (a) trend analysis of COVID-19 cases, (b) infection ratio (hot and cold spots) and Moran’s Index and (c) determinants based on regression.

The study needs to give attention to these corrections:

**Main comments:**

1) The Introduction section, Summary and Discussion, and Conclusion section need to be strengthened. Introduction section may be well connected with the other sections of the manuscript.
2) Trend analysis, infection ratio, hot and cold spots, and regression results are interpreted independently. Trend analysis may be interpreted for its plausible role of the concentration of COVID-19 cases in a few districts of India and infection ratio. Authors may explain what are the implications of manifold rise of recovery rates until July 2020 on the infection ratio or the implications of increase of average deceased cases by 734 times during studied period.
3) A large focus is given to explaining unadjusted beta coefficients from the results of regression model. Rather, the adjusted figures may be given more writing space. Independent variables are in percent in the linear regression model. Understanding that, the variables effects are interpreted in terms of levels. The interpretation of Table 2 may be revised.
4) In Table 2, authors may check the significance values and the respective confidence interval for unadjusted coefficients. These may be serious errors.
5) The manuscript may conclude strongly about the four determinants connecting it with the trend analysis, infection ratio and hot and cold spots.
6) The manuscript needs an English language editing and flow may be maintained.

**Minor comments:**

A) In abstract section, (1) the full form of COVID-19 disease may be written correctly, (2) whether authors have used prevalence rate from the crowdsourced data? (3) authors may specific reasons for using data from 14 March 2020, (4) authors may mention crucial information for policy discourse in the conclusion.
B) In Introduction section, (1) line no. 4-6: authors please check the sentence and growth rate of COVID-19 disease in the first three months from Jan to Mar 2020 perhaps, (2) line no. 8-10: check for definite measures, the reduction in doubling time is not apparent from Fig S1, and the second part of the same sentence is contradictory to the first part, (3) line no. 18-19: adults being less susceptible to disease compared to older is based on some assumptions, (4) line no. 22-23: Co-morbid situations are important but reference is missing, (5) line no. 26-27: under-five mortality and nutritional status children may not be associated with the *spread* of disease.
C) In Outcome variable section, (1) line no. 72, authors check formula.
D) In Spatial Analysis section, (1) line no. 111: description of “low-low” is not given in text.
E) In Result section, (1) line no. 114: authors may check the source used for data if they wish to write “every day since”, (2) line no. 116: Fig S2 and Table S1 provide the same results, (3) line no. 118-121: based on average bi-weekly and base numbers, authors may choose a different growth rate, also Table A1 is not found in text, (4) line no. 129-130: per one hundred thousand versus 100 thousand, authors may use same wording, (5) line no. 146-147: how one confirmed case and positive case are different? (6) line no. 193-194: the interpretation of likelihood in case of linear regression results, (7) line no. 195: which levels of infrastructure has higher effects is not clear from text and Table 2.
F) In Summary and Discussion section, (1) line no. 213: if demographic transition is an appropriate reason, then assumption need to be mentioned in this manuscript, (2) line no. 249: undefined term “unavoidable economic activities”, (3) line no. 259: study design may not be appropriate reason for not explaining co-morbidities in association with disease.
G) In Conclusion section, (1) line no. 267: understanding of disease only by Spatial distribution and its determinants is not supported by the analysis.
