# Supplementary material for: District level correlates of COVID-19 pandemic in India during March-October 2020
Source: PLoS One. 2021 Sep 30;16(9):e0257533. doi: 10.1371/journal.pone.0257533 (PMC8483309; doi:10.1371/journal.pone.0257533)
Supplement: Attachment — Submitted filename: Response to the reviewers.docx [file pone.0257533.s006.docx]

**Reviewer #1:**

Thank you for the opportunity to review this manuscript on a very topical issue. The overall idea, writing style and intent is good . The concerns regarding the study itself are however as below:

1. The study analyses data between march to July 2020 and therefore the timeliness of the publication and its findings itself may not resonate with the reader. Apart from the fact that things have changed since this study was done, the pandemic is now on the decline and in reality, widely prevalent in states like Kerala which did not need mention early in the pandemic given the State did so well.

**Reply: We have updated the study until 31^st^ October 2020 covering the first eight months of COVID-19 pandemic**.

2. It may be pertinent to revisit the timeline and consider maybe an analysis at least till the start of the winter months to make this a more relevant piece today.

**Reply: We agree with the reviewer that some of the dynamics of COVID-19 has changed now. Yet, the main findings of the study remains consistent when we carried out regression analysis on 31^st^ July and 31^st^ October 2020 separately.**

**The findings of the study is therefore relevant now and will be useful for future outbreak of other such diseases.**

3. The use of open source data , including NFHS is a good step, but NFHS data from next round too is now available.

**Reply: NFHS-5 data has not been released for many central and northern states yet.**

4. rather than only using above 60 age group, considering the middle age-groups - the working age groups may have also helped give important insights.

**Reply: We have included this variable in the revised analysis.**

5. The Sociodemographic variables did not include one on household income or occupation, while education may have filled some of that gap but skewed the findings.

**Reply: Household income or occupation data is NOT available at district level from Census or NSSO. Income data is often subject to reporting bias due to several reasons. To measure the economic status of districts, we have chosen proxy indicators of economic status, room/soap/water/toilet/.**

6. I was intrigued too by the selection of health indicators- fasting glucose , use of tobacco etc for women? Would this not under-represent prevalence across males of important co-variates like smoking? The access to healthcare is another important variable besides the consideration of pre-existing respiratory illness. A lot of selected variables related to immunisation and childhood illness and health indicators while this age group was largely the unaffected age group in the pandemic.

**Reply: The design of the regression analysis presented here is not an individual level of analysis, rather a group level (district level) analysis. Our independent variables are taken at macro level (i.e. at district level) to determine overall health status and health care service utilisation status. Therefore, fasting glucose or use of tobacco among women is representative to the overall health status of total population of the district. Similarly, immunisation and childhood illness is taken to represent overall health status or health care service utilisation.**

7.Line 283 - has an important spell edit - in terms of CoVID-19 protocols- not wearing the mask in pubic place to be edited to public place!

**Reply: We have corrected this.**

8. The overall intention of district-level correlates determining COVID-19 and feeding policy and management of the pandemic will be well-served if the right variables are selected for the analyses, and over a longer time window to help district-level planning and management.

**Reply: Thank you. We have updated the study for longer time window and we included the variable suggested by the reviewer**.

9. Use of the infection ratio is welcome but the correlates and determinants need addressing.

**Reply: We have analysed the correlates in table 2.**

10. Thank you for the clear and succinct writing style but do revisit some of the points addressed above.

**Reviewer 2**

The study on “District level correlates of COVID-19 pandemic in India” about exploring the hot and cold spots in terms of districts and finding the socio-economic, demographic and health-related determinants of COVID-19 disease in India. The study uses publicly available data from COVID19 India API. The study uses descriptive statistics of COVID-19 disease and linear regression model using indicators from NFHS-4 and Census of India to establish the findings. The outcomes of the study are based on three major findings from: (a) trend analysis of COVID-19 cases, (b) infection ratio (hot and cold spots) and Moran’s Index and (c) determinants based on regression.

The study needs to give attention to these corrections:

**Main comments:**

1) The Introduction section, Summary and Discussion, and Conclusion section need to be strengthened. Introduction section may be well connected with the other sections of the manuscript.

**Reply: We updated the introduction, discuss and conclusion.**

2) Trend analysis, infection ratio, hot and cold spots, and regression results are interpreted independently. Trend analysis may be interpreted for its plausible role of the concentration of COVID-19 cases in a few districts of India and infection ratio.

Authors may explain what are the implications of manifold rise of recovery rates until July 2020 on the infection ratio or the implications of increase of average deceased cases by 734 times during studied period.

**Reply: We have updated and interpreted the results as per the suggestion of the reviewer. There is no impact of recovered and death cases on infection ratio. The infection ratio includes all the confirmed cases (including the dead and recovered cases).**

3) A large focus is given to explaining unadjusted beta coefficients from the results of regression model. Rather, the adjusted figures may be given more writing space. Independent variables are in percent in the linear regression model. Understanding that, the variables effects are interpreted in terms of levels. The interpretation of Table 2 may be revised.

**Reply: In the revised analysis, we reduced the interpretation for unadjusted beta coefficients.**

4) In Table 2, authors may check the significance values and the respective confidence interval for unadjusted coefficients. These may be serious errors.

**Reply: In earlier table, there was exchange of “comma” and “decimal”. In the revised draft, we have corrected it.**

The manuscript may conclude strongly about the four determinants connecting it with the trend analysis, infection ratio and hot and cold spots.

**Reply: We have revised the conclusion.**

5) The manuscript needs an English language editing and flow may be maintained.

**Reply: We have revised the conclusion**

**Minor comments:**

A) In abstract section, (1) the full form of COVID-19 disease may be written correctly, (2) whether authors have used prevalence rate from the crowdsourced data? (3) authors may specific reasons for using data from 14 March 2020, (4) authors may mention crucial information for policy discourse in the conclusion.

**Reply: We have included “Corona Virus Disease 2019” in the abstract. We have calculated “Infection ratio” which is mentioned in the abstract.**

B) In Introduction section, (1) line no. 4-6: authors, please check the sentence and growth rate of COVID-19 disease in the first three months from Jan to Mar 2020 perhaps,

**Reply: We have modified this sentence.**

C) (2) line no. 8-10: check for definite measures, the reduction in doubling time is not apparent from Fig S1, and the second part of the same sentence is contradictory to the first part.

**Reply: We have modified the explanation.**

(3) line no. 18-19: adults being less susceptible to disease compared to older is based on some assumptions,

**Reply: We gave the reference of this statement.**

(4) line no. 22-23: Co-morbid situations are important but reference is missing, (5) line no. 26-27: under-five mortality and nutritional status children may not be associated with the *spread* of disease.

**Reply: We have included Acharya and Porwal 2020.**

**For other point see above reply 6 to the the first reviewer.**

D) In Outcome variable section, (1) line no. 72, authors check formula.

**Reply: Thank you. The divide symbol was missing while transporting the word file to PDF file. Corrected it now**

E) In Spatial Analysis section, (1) line no. 111: description of “low-low” is not given in text.

**Reply: We added this description in the revised manuscript.**

In Result section, (1) line no. 114: authors may check the source used for data if they wish to write “every day since”,

**Reply: We modified according to the reviewer’s suggestion.**

(2) line no. 116: Fig S2 and Table S1 provide the same results,

**Reply: We have modified the relevant sentence.**

(3) line no. 118-121: based on average bi-weekly and base numbers, authors may choose a different growth rate,

**Reply: We want to retain it as it is easy for interpretation.**

also Table A1 is not found in text,

**Reply: We explain table S1 in the revised manuscript.**

(4) line no. 129-130: per one hundred thousand versus 100 thousand, authors may use same wording.

**Reply: Addressed throughout the manuscript as per 100, 000 population.**

(5) line no. 146-147: how one confirmed case and positive case are different?

**Reply: They are same. We now use only the term “confirmed case” throughout the paper.**

(6) line no. 193-194: the interpretation of likelihood in case of linear regression results,

**Reply: We have modified this section.**

(7) line no. 195: which levels of infrastructure has higher effects is not clear from text and Table 2.

**Reply: Addressed in the interpretation section of table 2**

F) In Summary and Discussion section, (1) line no. 213: if demographic transition is an appropriate reason, then assumption need to be mentioned in this manuscript,

**Reply: We have modified this sentence.**

(2) line no. 249: undefined term “unavoidable economic activities”,

**Reply: We added some examples.**

(3) line no. 259: study design may not be appropriate reason for not explaining co-morbidities in association with disease.

**Reply: We have modified this sentence.**

G) In Conclusion section, (1) line no. 267: understanding of disease only by Spatial distribution and its determinants is not supported by the analysis.

**Reply: We politely disagree with the reviewer in this point. Our analysis identified COVID-19’s spatial distribution (through maps, Moran I and LISA cluster map). We also carried out regression analysis to identify the correlates of COVID-19 at district level.**

**We have replaced the word “determinants” by “correlates”.**
