# Supplementary material for: District level correlates of COVID-19 pandemic in India during March-October 2020
Source: PLoS One. 2021 Sep 30;16(9):e0257533. doi: 10.1371/journal.pone.0257533 (PMC8483309; doi:10.1371/journal.pone.0257533)
Supplement: Attachment — Submitted filename: Review_PLOSONE_34338_2.docx [file pone.0257533.s007.docx]

The study on “District level correlates of COVID-19 pandemic in India” about exploring the hot and cold spots in terms of districts and finding the socio-economic, demographic and health-related determinants of COVID-19 disease in India has remained more or less at the same position.

Authors may consider these suggestions for future works.

1) The result section is crowded with minor small descriptions such as data description of recovery rates, death rates, which is nowhere connected with any succeeding results, state level and then district level infection ratios, and then again descriptive statistics as shown in Table 1 from which basic statistics for many considered variables are described.
2) The regression results are very poorly summarised. Researchers have ignored the importance of adjusted over unadjusted models. The results clearly warn through the change in P values and coefficients while showing the case of unadjusted and adjusted models. It is percent population in 15-59 years, population density, % urban population, testing, and stunting which are outcomes from regression analysis but not adequately explained in result as well as discussion section of the manuscript.
3) The introduction should be updated with more evidences from the works of literature. The transmission rate of virus of COVID-19 disease is higher during initial times whereas authors have considered it slow based on some works of literature. The widespread of media coverage are not supported by good references. These are controversial and dithering statements given the pandemic.
4) Authors have not taken the numbers and its interpretation seriously. There is ignorance on the part of authors.
5) The term bi-weekly is cumulative or point COVID-19 cases, it is not clear in the text. The bi-weekly numbers of 11 lakhs are shown in Appendix S1 which reveals the bi-weekly peak, as terminology used by authors, was 1244430 in the next week in contrast to the previous week mentioned in text. The Figure 1 and Table S1 shows average numbers; this bi-weekly peak/number is not a meaningful description when original COVID19 data from the API itself provides a more robust measurement.
6) The percent of women whose husbands are away for the last six months is taken as the proxy of out-migration but not taken as one of the variables in analysis. However, percent of marginal workers is considered with the same max and min values of that variable as explained in text.
7) Again, ‘positive (absolute values)’ wording is used in the panel A in Fig 2 and 3. Legends of the maps shown in Fig 2 are inconsistent with Fig 3.
8) The write-up needs to be much more scientific and richer in content which is lacking throughout.
