# Supplementary material for: District level correlates of COVID-19 pandemic in India during March-October 2020
Source: PLoS One. 2021 Sep 30;16(9):e0257533. doi: 10.1371/journal.pone.0257533 (PMC8483309; doi:10.1371/journal.pone.0257533)
Supplement: Attachment — Submitted filename: Reply to the reviewers 31 Aug 2021.docx [file pone.0257533.s008.docx]

The study on “District level correlates of COVID-19 pandemic in India” about exploring the hot and cold spots in terms of districts and finding the socio-economic, demographic and health-related determinants of COVID-19 disease in India has remained more or less at the same position.

**Reviewer comments:**

Authors may consider these suggestions for future works.

The result section is crowded with minor small descriptions such as data description of recovery rates, death rates, which is nowhere connected with any succeeding results, state level and then district level infection ratios, and then again descriptive statistics as shown in Table 1 from which basic statistics for many considered variables are described.

**Response – Thank you. We agree with the reviewer that we have done sufficient analysis to explore district level COVID-19 pattern. Therefore, we have modified our objective (Line 116-118). We kept the national level analysis (Figure 1) as background information. The state level analysis is presented only in the appendix with a short description of these results in line number 216-228.**

The regression results are very poorly summarised. Researchers have ignored the importance of adjusted over unadjusted models. The results clearly warn through the change in P values and coefficients while showing the case of unadjusted and adjusted models. It is percent population in 15-59 years, population density, % urban population, testing, and stunting which are outcomes from regression analysis but not adequately explained in result as well as discussion section of the manuscript.

**Response – We have updated regression results (the unadjusted model in the line number 308-325 and then the adjusted model from 326 to 341 of the manuscript. All the significant variables were explained adequately in the revised manuscript.**

The introduction should be updated with more evidences from the works of literature. The transmission rate of virus of COVID-19 disease is higher during initial times whereas authors have considered it slow based on some works of literature. The widespread of media coverage are not supported by good references. These are controversial and dithering statements given the pandemic.

**Response – We have updated the introduction section. We have not refered any media article; we rather refered articles by researchers. Due to nation-wide lockdown, the transmission rate was slow at the beginning of COVID19 in India. We have also included some good references.**

Authors have not taken the numbers and its interpretation seriously. There is ignorance on the part of authors.

**Response – We have revised the write-up in the revised draft.**

The term bi-weekly is cumulative or point COVID-19 cases, it is not clear in the text. The bi-weekly numbers of 11 lakhs are shown in Appendix S1 which reveals the bi-weekly peak, as terminology used by authors, was 1244430 in the next week in contrast to the previous week mentioned in text. The Figure 1 and Table S1 shows average numbers; this bi-weekly peak/number is not a meaningful description when original COVID19 data from the API itself provides a more robust measurement.

**Response – The term bi-weekly is non-cumulative. The number is correct and text was modified accordingly. The numbers have been revised in the text (line number 83-85 of the manuscript). The term bi-weekly in Figure 1 and Table S1 was not cumulative cases. It showed the average number of cases per 14 days. We showed 14 days average cases as when we present per each day, it becomes relatively cumbersome.**

The percent of women whose husbands are away for the last six months is taken as the proxy of out-migration but not taken as one of the variables in analysis. However, percent of marginal workers is considered with the same max and min values of that variable as explained in text.

**Response – We have removed the proxy of out-migration variable (the percent of women whose husbands are away for the last six months) from tables and text as it has not been included in regression analysis.**

Again, ‘positive (absolute values)’ wording is used in the panel A in Fig 2 and 3. Legends of the maps shown in Fig 2 are inconsistent with Fig 3.

**Response – We have revised this legend by “COVID19 Confirmed” and used the “Absolute number of COVID-19 cases” in panel A of both Figure 2 & 3.**

The write-up needs to be much more scientific and richer in content which is lacking throughout.

**Response: We have modified the write-up.**

**Reviewer #3:** The study is important, interesting and generally well written. I have some small recommendations which should be addressed before it can be published.

Comments

1. This study clearly states that its analysis focuses on the months between July and October 2020. Clearly the covid-19 pandemic is ever-changing and India is clearly in the midst of a catastrophic second wave. The authors have clearly put much effort into their paper so to expect them to include data beyond October 2020 is unreasonable. Instead, the authors should provide a comparison between the first and second waves in their discussion - perhaps comparing case numbers in districts during the first wave to the second? Perhaps discussing which of the factors discussed in this paper may have contributed to this devastating second wave?

**Response ‑If we discuss first and second wave of COVID-19 together, we need to discuss it based on analysis, speculations will not be a good strategy. However, adding new analysis for the second wave in this study is beyond the scope of the paper. Covid-19 situation has been ever changing, and hence we want to restrict our analysis within the first wave.**

**Yet, as per reviewer’s suggestion, we compared the situation in the second wave as a part of discussion (Line number 402-424 of the manuscript).**

2. As with a fellow reviewer I'm confused by the use of health-related variables focussing only on women (% women with diabetes, % women with cancer, % women consuming tobacco etc). As men are at greater risk of covid-19 why have they been excluded from these analyses? Why also are socioeconomic variables such as religion (% Hindu) used? There may be good reasons for this, however the manuscript would benefit from the explanation of such reasons.

**Response – The design of the regression analysis presented here is not an individual level of analysis, rather a group level (district level analysis. Our independent variables are taken at macro level (i.e., district level) to determine overall health status and health care service utilization status. Therefore, fasting glucose or use of tobacco among women is representation to the overall health status of total population of the district. Similarly, immunization and childhood illness are taken to represent overall health status and health care service utilization. Percent of Hindu population is used as an indicator of majority population (which indirectly reflect the presence of minority population) at district level.**

3. The manuscript makes no mention of the causative agent of covid-19, SARS-CoV-2. At least one sentence explaining that SARS-CoV-2 is the virus whilst covid-19 is the disease caused by the virus. Furthermore, the authors must make an effort to discriminate SARS-CoV-2 and covid-19 when discussing spread. For instance, the authors say "COVID-19 has been spreading rapidly in the urban areas". This is inaccurate, SARS-CoV-2 has been spreading.

**Response – We have mentioned SARS-CoV-2 in the introductory part of the revised manuscript (Line no. 70-73**
